# Association of sperm DNA fragmentation index with semen quality and ART outcomes: real-world evidence from a retrospective cohort

**DOI:** 10.1186/s12610-026-00308-2

**Published:** 2026-03-20

**Authors:** Chaofeng Wei, Qihui Liang, Danqi Liu, Yi Yu, Yihe Wei, Yuan Li, Haicui Wu, Fang Lian

**Affiliations:** 1 First College of Clinical Medicine, Shandong University of Traditional Chinese Medicine, Jinan, China; 2 Department of Obstetrics and Gynecology, BC Children’s Hospital Research Institute, University of British Columbia, Vancouver, BC V5Z 4H4 Canada; 3 Traditional Chinese Medicine department, International Peace Maternity and Child Health Hospital of China welfare institute, Shanghai, China; 4 Integrative Medicine Research Centre of Reproduction and Heredity, The Affiliated Hospital of Shandong University of Traditional Chinese Medicine, Jinan, China

**Keywords:** Male infertility, Sperm DNA fragmentation index, Assisted reproductive technology, Semen quality, Infertilité masculine, Indice de Fragmentation de l’ADN spermatique, Technique de Procréation médicalement assistée, Qualité du Sperme

## Abstract

**Background:**

Sperm DNA fragmentation index (DFI) has emerged as a potential marker of male fertility, but its clinical value in assisted reproductive technology (ART) remains debated. This study aimed to investigate the association between sperm DFI and conventional semen parameters, embryological outcomes, and pregnancy outcomes in ART, using a large real-world dataset.

**Results:**

A total of 1,784 ART cycles performed at a single center between January 2020 and January 2022 were analyzed. Patients were categorized into normal (< 30%) and high (≥ 30%) DFI groups. Compared with the normal DFI group, men with high DFI had significantly lower sperm concentration (B = − 31.15, 95% CI: − 44.75 to − 17.55), progressive motility (B = − 12.21, 95% CI: − 14.87 to − 9.56), and total motility (B = − 18.00, 95% CI: − 21.38 to − 14.62; all *P* < 0.001). After adjusting for female and male factors, high DFI was associated with a significantly lower 2PN fertilization rate (β = − 0.257, 95% CI: − 0.491 to − 0.024, *P* = 0.031), while no significant differences were observed in top-quality embryo rate, transferable embryo rate, or pregnancy outcomes. Sensitivity analyses using alternative statistical methods (ZOIB and PSM) and varying DFI thresholds (25% and 35%) yielded results completely consistent with the main analyses.

**Conclusions:**

High sperm DFI is independently associated with poorer semen quality and a reduced 2PN fertilization rate but was not significantly associated with embryo quality or pregnancy outcomes in this study. These findings support the clinical relevance of DFI in evaluating male fertility potential while suggesting its limited predictive value for ART success when female and cycle-related factors are considered.

**Supplementary Information:**

The online version contains supplementary material available at 10.1186/s12610-026-00308-2.

## Résumé

**Contexte:**

L’indice de fragmentation de l’ADN spermatique (DFI) est apparu comme un marqueur potentiel de la fertilité masculine, mais sa valeur clinique en cas de technique de procréation médicalement assistée (PMA) reste débattue. La présente étude visait à étudier l’association entre le DFI spermatique et les paramètres conventionnels du sperme, les résultats embryologiques et les résultats de grossesse en PMA, à partir d’un vaste ensemble de données réelles.

**Résultats:**

Un total de 1784 cycles de PMA réalisés dans un même centre entre janvier 2020 et janvier 2022 ont été analysés. Les patients ont été classés en groupes DFI normaux (<30 %) et élevés (≥30 %). Comparés au groupe DFI normal, les hommes avec un DFI élevé présentaient une concentration de spermatozoïdes significativement plus faible (B = –31,15 ; IC 95 % : –44,75 à –17,55), une mobilité progressive (B = –12,21, IC 95 % : –14,87 à –9,56) et une motilité totale (B = –18,00, IC 95 % : –21,38 à –14,62 ; tous *P* < 0,001). Après ajustement pour les facteurs féminins et masculins, un DFI élevé était associé à un taux de fécondation significativement plus faible à 2PN (β = –0,257, IC 95 % : –0,491 à –0,024, *P* = 0,031), tandis qu’aucune différence significative n’était observée pour le taux d’embryons de haute qualité, le taux d’embryons transférables ou les résultats de la grossesse. Les analyses de sensibilité utilisant des méthodes statistiques alternatives (ZOIB et PSM) et des seuils DFI variables (25 % et 35 %) ont donné des résultats totalement cohérents avec les analyses principales.

**Conclusions:**

Un DFI spermatique élevé est indépendamment associé à une qualité de sperme inférieure et à un taux de fécondation à 2PN réduit, mais cela n’a pas été significativement associé à la qualité embryonnaire ou aux résultats de la grossesse dans cette étude. Ces résultats soutiennent la pertinence clinique du DFI dans l’évaluation du potentiel de fécondité masculine, tout en suggérant sa valeur prédictive limitée pour le succès de la PMA lorsque les facteurs féminins et liés au cycle sont pris en compte.

## Introduction

The global incidence of infertility continues to rise, affecting approximately 8–12% of couples worldwide [1]. In China, shifting attitudes among younger couples toward marriage and childbearing, combined with the implementation of the two-child and three-child policies, have led to a significant proportion of advanced-age individuals within the infertile population, As a result, the demand for assisted reproductive technology (ART) has risen rapidly. By the end of 2017, the number of ART cycles in China had reached 1.15 million, and by 2019, 517 ART centers were established across mainland China [2].

About half of infertility cases are attributed to male factors [1], which warrants serious attention. Notably, data from successive editions of the *World Health Organization (WHO) Laboratory Manual for the Examination and Processing of Human Semen* (hereinafter referred to as “the Manual”) have demonstrated a progressive decline in semen quality over the years. For example, the first edition, published in 1980, defined normal sperm concentration as 60 × 10⁶/mL [3], whereas the sixth edition, released in 2021, reduced this threshold to 15–18 × 10⁶ /mL [4]. It is important to assess male fertility correctly so that effective interventions can be made in a timely manner.

Traditionally, male fertility has been assessed primarily through routine semen analysis, which includes parameters such as semen volume, sperm concentration, morphology, and motility. However, this approach has limitations and may not fully reflect a man’s reproductive potential. For instance, according to WHO standards [5], a significant proportion of men with proven fertility—such as those undergoing vasectomy—may still exhibit low sperm motility and abnormal morphology [6]. Moreover, some men with normal semen parameters may nonetheless experience infertility [7]. These observations suggest that additional molecular-level factors, beyond conventional semen analysis, may play a critical role in determining male fertility.

In recent years, some new assessments such as the sperm DNA Fragmentation Index (DFI) have emerged as valuable clinical tools for evaluating male reproductive potential. The 6th edition of the Manual clearly states that DFI testing is an important supplement to standard semen analysis [4]. This parameter advances semen evaluation from traditional microscopy to the molecular level. DNA fragmentation is the result of DNA strand breaks that occur during spermatogenesis and up to maturation due to various unfavorable factors such as poor lifestyle, systemic diseases, or genital tract infections. Commonly used DFI assays include terminal deoxynucleotidyl transferase dUTP nick-end labeling (TUNEL), single-cell gel electrophoresis (COMET), sperm chromatin structure analysis (SCSA), and sperm chromatin dispersion (SCD). Among these, SCSA, first described by Evenson et al., is an indirect approach for assessing DNA damage [8], favored for its high reproducibility [9].

DFI reflects the integrity of sperm DNA and the extent of damage. Elevated DFI has been associated with reduced natural conception [10] and ART success rates [11]. In contrast, some studies reported no significant effect of DFI on pregnancy outcomes in IVF or ICSI cycles, regardless of whether autologous or donor oocytes are used [12]. Given the conflicting findings, some guidelines do not currently recommend routine DFI testing in infertility evaluations. This study therefore aimed to investigate the association between DFI and conventional semen parameters. Furthermore, we assessed the relationship between DFI and both laboratory and clinical outcomes, while rigorously controlling for potential confounders.

## Materials and methods

### Study population

This retrospective cohort study utilized data from patients who underwent IVF/ICSI-ET at the Reproduction and Genetics Center of the Affiliated Hospital of Shandong University of Traditional Chinese Medicine between January 2020 and January 2022, as recorded in the Reproductive Case Management System. Eligible cycles were those in which the male partner had undergone sperm DFI testing. Exclusion criteria included chromosomal abnormalities; polycystic ovary syndrome (PCOS); endometriosis; recurrent miscarriages or repeated implantation failures; uterine malformations (e.g., bicornuate and unicornuate uterus); endocrine diseases (e.g., diabetes, thyroid disease); and mixed IVF+ICSI or rescue-ICSI (R-ICSI) cycles. Patients were grouped based on DFI levels (< 30% vs. ≥30%).

**Fig. 1:**
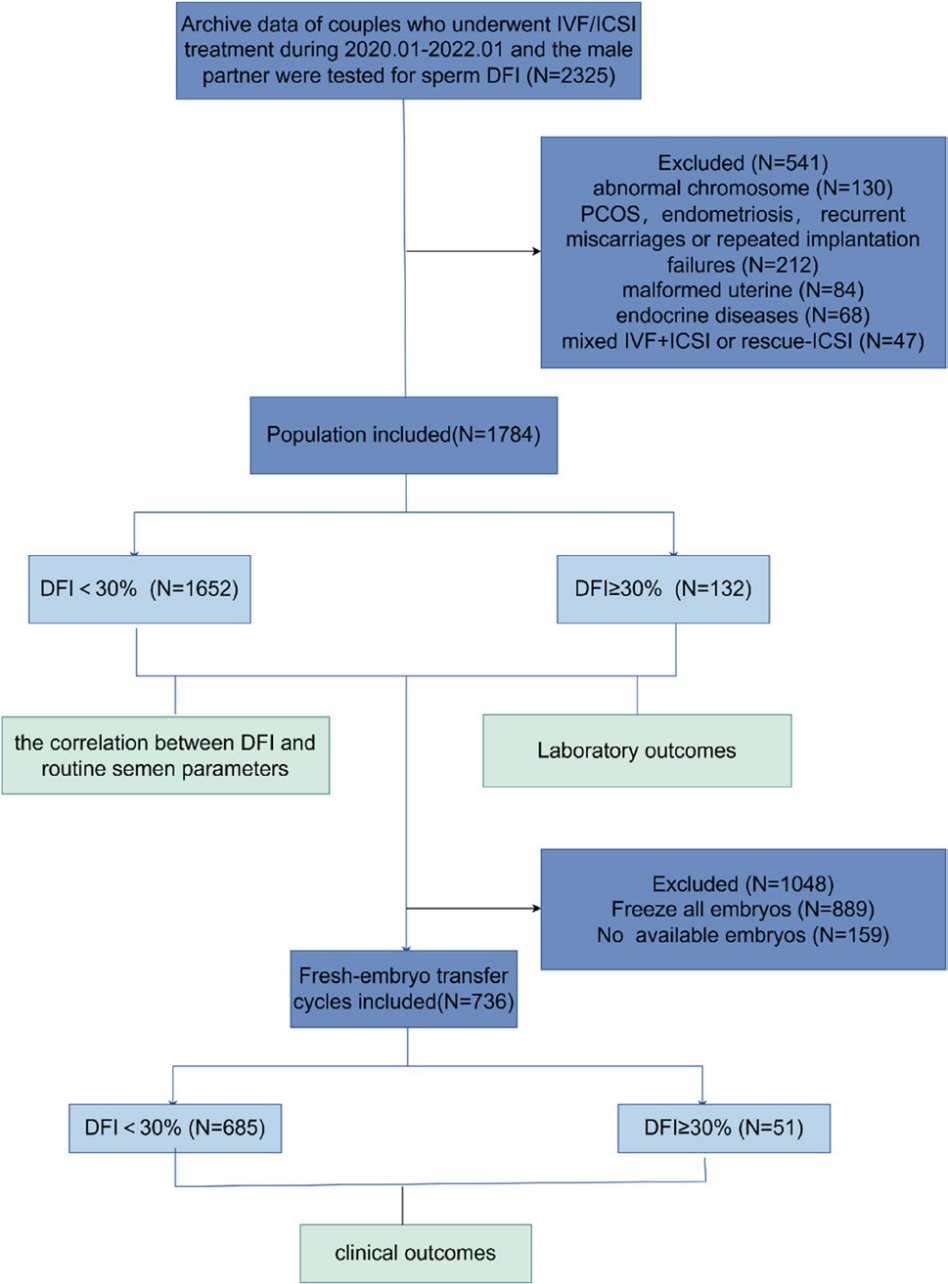
Flowchart of the inclusion and exclusion process

Analyses proceeded in three stages: (1) assessing the correlation between DFI and semen parameters; (2) comparing embryological outcomes by DFI group; and (3) evaluating pregnancy outcomes in fresh embryo transfer cycles to avoid confounding from embryo freezing and thawed transfer strategies. The inclusion process is illustrated in Fig. 1.

### Semen collection

The patient should abstain for 2–7 days, collecting all semen using masturbation into a sterile, dry disposable plastic specimen cup, which is then placed in a 37 °C incubator. Once the specimen has completely liquefied, a semen quality analysis should be completed within 1 h. Additionally, the semen sample should be aliquoted into EP tubes and stored in a -20 °C freezer for future sperm DFI assessment, to be completed within one week.

### Conventional semen analysis

According to the operational requirements outlined in the 5th edition of the Manual [13], once the semen has fully liquefied, laboratory technicians assess the appearance, volume, viscosity, liquefaction time, and pH of the semen. A 3 µL aliquot of the well-mixed semen sample is deposited onto a disposable sperm counting chamber (SAS Medical, SAS) and analyzed using a computer-assisted sperm analysis system (SAS) with the SAS II ^®^ version 2.3 software to determine the total sperm count, concentration, and total sperm motility (TSM, TSM = PR + NP), including progressive motility (PR) and non-progressive motility (NP). Sperm morphology analysis is performed manually; 5 µL of the fully liquefied semen specimen is placed on a slide, and a smear is prepared using a spreading technique. After allowing it to air dry and fixing it, sperm are stained using Diff-Quick staining reagents (Zhuhai Beiso Company) and analyzed under an optical microscope for morphology. More than 200 sperm per sample are counted and assessed to calculate the percentage of normally shaped sperm. In cases of excessively high sperm concentration, samples should be diluted and centrifuged before analysis to prevent errors due to high-frequency collisions.

### DFI testing (SCSA method)

The sperm DFI was assessed using the SCSA method via acridine orange (AO) staining and flow cytometry. Cryopreserved semen samples were thawed in a 37 °C water bath and immediately diluted with TNE buffer (0.01 M Tris-HCl, 0.15 M NaCl, 1 mM EDTA) to a final concentration of 1-2 × 10^6^ sperm/mL. Sperm were then treated with an acid-detergent solution (pH 1.2) for 30 s to induce partial denaturation of DNA at sites of fragmentation, followed by staining with an AO-containing buffer (Guowei Biotechnology Co., Ltd.). Samples were analyzed using a BD FACSCanto II flow cytometer (BD Biosciences). For each sample, at least 5,000 sperm cells were captured, and measurements were performed at least twice to ensure reproducibility. The gating strategy followed established protocols to exclude debris and non-sperm cells, with the DFI calculated as the ratio of red (denatured, single-stranded DNA) to total (red + green) fluorescence intensity.

Regarding sample stability, previous validation studies have demonstrated that semen samples stored at -20℃ for up to 7 days maintain stable DFI levels, with original seminal plasma providing a protective antioxidant buffering system that minimizes oxidative stress-induced damage [14]. Intra-laboratory quality control was maintained using aliquoted frozen reference samples, and the laboratory regularly participates in external quality assessment (EQA) programs to ensure technical consistency.

### Outcome indicators and calculation methods

The 2PN fertilization rate for each cycle was calculated as the number of normally fertilized oocytes (with two pronuclei, 2PN) divided by the total number of retrieved oocytes. Day 3 embryos were evaluated based on the Istanbul consensus [15]. Embryos with a score ≥ 7 (Grade I) were considered top-quality, while those with a score ≥ 6 (Grade II or above) were defined as transferable. Accordingly, the transferable embryo rate was calculated for each cycle as the number of transferable embryos divided by the total number of normally fertilized oocytes (2PN). Biochemical pregnancy was defined as a positive β-human chorionic gonadotropin (β-HCG) result (≥ 25 IU/L) 14 days after embryo transfer. Clinical pregnancy was defined as the presence of at least one intrauterine gestational sac with heartbeat, observed 4–6 weeks after embryo transfer. Pregnancy loss was defined as any occurrence of spontaneous or induced abortion after confirmation of clinical pregnancy. The pregnancy loss rate (PLR) was calculated as: (number of pregnancy loss cycles / number of clinical pregnancy cycles) × 100%. Ectopic pregnancy was defined as a pregnancy occurring outside the uterine cavity. Live birth was defined as the delivery of at least one live infant per embryo transfer cycle.

### Statistical methods

All analyses were conducted using SPSS 26.0 and R software (version 4.4.1). A two-sided *P* value of < 0.05 was considered statistically significant.

#### Descriptive statistics and baseline comparisons

For continuous variables, normality was assessed using histograms and the Shapiro–Wilk test. Normally or approximately normally distributed variables were expressed as mean ± standard deviation (SD) and compared using Student’s *t*-test. Skewed variables were reported as median (25th, 75th percentile) and compared using the Mann–Whitney *U* test. Categorical variables were summarized as frequencies (%) and compared using Pearson’s chi-square test or Fisher’s exact test, as appropriate. Sperm morphology data exhibited a missing rate of approximately 29%. To assess whether the missing data were related to baseline semen quality, descriptive characteristics were compared between participants with and without available morphology data.

#### Linear regression analysis of semen parameters

Simple linear regression was first used to analyze the association between sperm DFI and conventional semen parameters. Subsequently, multivariate linear regression was applied to control for potential confounding factors. For sperm morphology, given the missing data, Multiple Imputation by Chained Equations (MICE) was employed to generate 5 imputed datasets prior to conducting the multivariate linear regression on the pooled data.

#### Multivariable regression analysis of embryological and pregnancy outcomes

The association between sperm DFI and the 2PN fertilization rate was examined using beta regression, which is appropriate for continuous proportions bounded between 0 and 1. To accommodate boundary values (0 and 1), a standard shrinkage transformation was applied using the formula y* = (y × (n – 1) + ε) / n, where ε = 0.0001 and n is the sample size, as recommended by Smithson and Verkuilen [16].

For other embryological outcomes, the presence of top-quality embryos was dichotomized as “yes” if at least one top-quality embryo was present; transferable embryo rate was categorized as “high” if > 50% of embryos were transferable. These binary outcomes, as well as pregnancy outcomes including biochemical pregnancy, clinical pregnancy, live birth, pregnancy loss, and ectopic pregnancy, were analyzed using multivariable logistic regression. Models were adjusted for clinically relevant covariates and for potential confounders identified by baseline differences (*P* < 0.05) between groups.

To further investigate the potential non-linear relationship between DFI as a continuous variable and reproductive outcomes, Restricted Cubic Spline (RCS) regression with four knots was performed. All models were adjusted for the same covariates used in the multivariable analysis. Non-linearity was assessed using the Wald test.

#### Sensitivity analyses

A sensitivity analysis was conducted using different methods tailored to the type of outcome to validate the robustness of the main findings. For embryological outcomes expressed as proportions, zero-one inflated beta (ZOIB) regression was used to accommodate boundary values (0 and 1) and distributional skewness. For pregnancy outcomes, a 1:3 nearest-neighbor propensity score matching (PSM) was performed using a set of clinically relevant baseline covariates, followed by logistic regression. Forest plots were used to summarize and visualize effect estimates (β or OR) and 95% confidence intervals (CIs) from both main and sensitivity analyses. Furthermore, to rigorously validate the robustness of the ≥ 30% DFI threshold, additional sensitivity analyses were performed using alternative DFI cut-offs of 25% and 35%. The associations between DFI and key reproductive outcomes (2PN fertilization rate, clinical pregnancy, and live birth) were re-evaluated using the same multivariable regression models as in the primary analysis.

## Results

A total of 1784 couples undergoing their first IVF/ICSI cycles were included to analyze the relationship between DFI and conventional semen parameters. Of these, 1,652 samples exhibited normal DFI, while 132 showed high DFI. After controlling for potential confounders related to couple characteristics and ART cycle parameters, we examined the association between DFI and laboratory outcomes. Following exclusion of cycles without transferable embryos or involving full embryo cryopreservation, 736 fresh embryo transfer cycles (685 normal DFI, 51 high DFI) were analyzed for pregnancy outcomes. Detailed results are presented below.

### Correlation between sperm DFI and conventional semen parameters

Comparisons between patients with and without available morphology data showed no significant differences in baseline characteristics, confirming that the missingness was random (Supplementary Table S1). Male baseline characteristics showed no significant differences in body mass index (BMI), infertility duration, infertility type, abstinence days, or blood type between groups (Table 1). However, the high DFI group had older males (36.89 vs. 34.58 years, *P* < 0.001), smaller testicular volumes (bilateral median: 15.0 vs. 18.0 mL, *P* = 0.003), and higher varicocele prevalence (absence of varicocele: 77.27% vs. 85.65%; *P* = 0.031). Semen quality was significantly impaired in the high DFI group, with reduced sperm concentration (43.55 vs. 77.40 × 10⁶/mL, *P* < 0.001), progressive motility (PR: 25.65% vs. 39.20%; *P* < 0.001), and total sperm motility (TSM: 35.80% vs. 55.49%, *P* < 0.001). In contrast, neither sperm morphology nor semen volume differed significantly between the high and normal DFI groups.

**Table 1:** Baseline characteristics and semen parameters of male participants by DFI group

Variable	Total (*n* = 1784)	DFI<30% (*n* = 1652)	DFI ≥ 30% (*n* = 132)	Statistic	*P*
DFI (%)	15.68 ± 9.50	13.78 ± 6.56	39.44 ± 8.53	t=-33.778	< 0.001^*^
Male age (year)	34.75 ± 5.68	34.58 ± 5.57	36.89 ± 6.49	t=-3.968	< 0.001^*^
Male BMI (kg/m^2^)	26.23 ± 4.04	26.18 ± 4.03	26.80 ± 4.14	t=-1.683	0.093
Infertility duration (year)	3.00 (2.00–5.00)	3.00 (2.00–5.00)	3.00 (2.00–5.00)	Z = 1.634	0.107
Infertility type, n (%)				χ²=0.321	0.571
Primary infertility	769 (43.11)	709 (42.92)	60 (45.45)		
Secondary infertility	1015 (56.89)	943 (57.08)	72 (54.55)		
Abstinence days (day)	5.00 (3.00–7.00)	4.00 (3.00–7.00)	5.00 (3.00–7.00)	Z = 1.125	0.267
Right testis volume (mL)	18.00 (15.00–20.00)	18.00 (15.00–20.00)	15.00 (15.00–20.00)	Z = 3.101	0.003^*^
Left testis volume (mL)	18.00 (15.00–20.00)	18.00 (15.00–20.00)	15.00 (15.00–20.00)	Z = 3.082	0.003^*^
Varicocele status, n (%)				χ²=8.860	0.031^*^
Normal	1517 (85.03)	1415 (85.65)	102 (77.27)		
Grade I	98 (5.49)	84 (5.08)	14 (10.61)		
Grade II	51 (2.86)	46 (2.78)	5 (3.79)		
Grade III	118 (6.61)	107 (6.48)	11 (8.33)		
Male blood type, n (%)				χ²=0.655	0.884
A	467 (27.04)	433 (27.10)	34 (26.36)		
B	575 (33.29)	528 (33.04)	47 (36.43)		
AB	189 (10.94)	176 (11.01)	13 (10.08)		
O	496 (28.72)	461 (28.85)	35 (27.13)		
Sperm volume (mL)	3.70 ± 1.48	3.69 ± 1.45	3.93 ± 1.74	t=-1.555	0.122
Sperm concentration (x10^6^/mL)	74.80 (40.05–127.40)	77.40 (42.00–130.80)	43.55 (21.50–79.62)	Z = 6.378	< 0.001^*^
PR (%)	38.19 ± 15.44	39.20 ± 15.04	25.65 ± 14.86	t = 9.968	< 0.001^*^
TSM, PR + NP (%)	54.03 ± 19.93	55.49 ± 19.34	35.80 ± 18.19	t = 11.303	< 0.001
Normal morphology	3.50 (2.00, 5.00)	3.50 (2.00, 5.00)	3.50 (2.00, 5.50)	Z=-0.58	0.564

*DFI* DNA fragmentation index, *PR* progressive motility, *NP* non-progressive motility, *TSM* total sperm motility

^*^Indicates *P* < 0.05

**Fig. 2:**
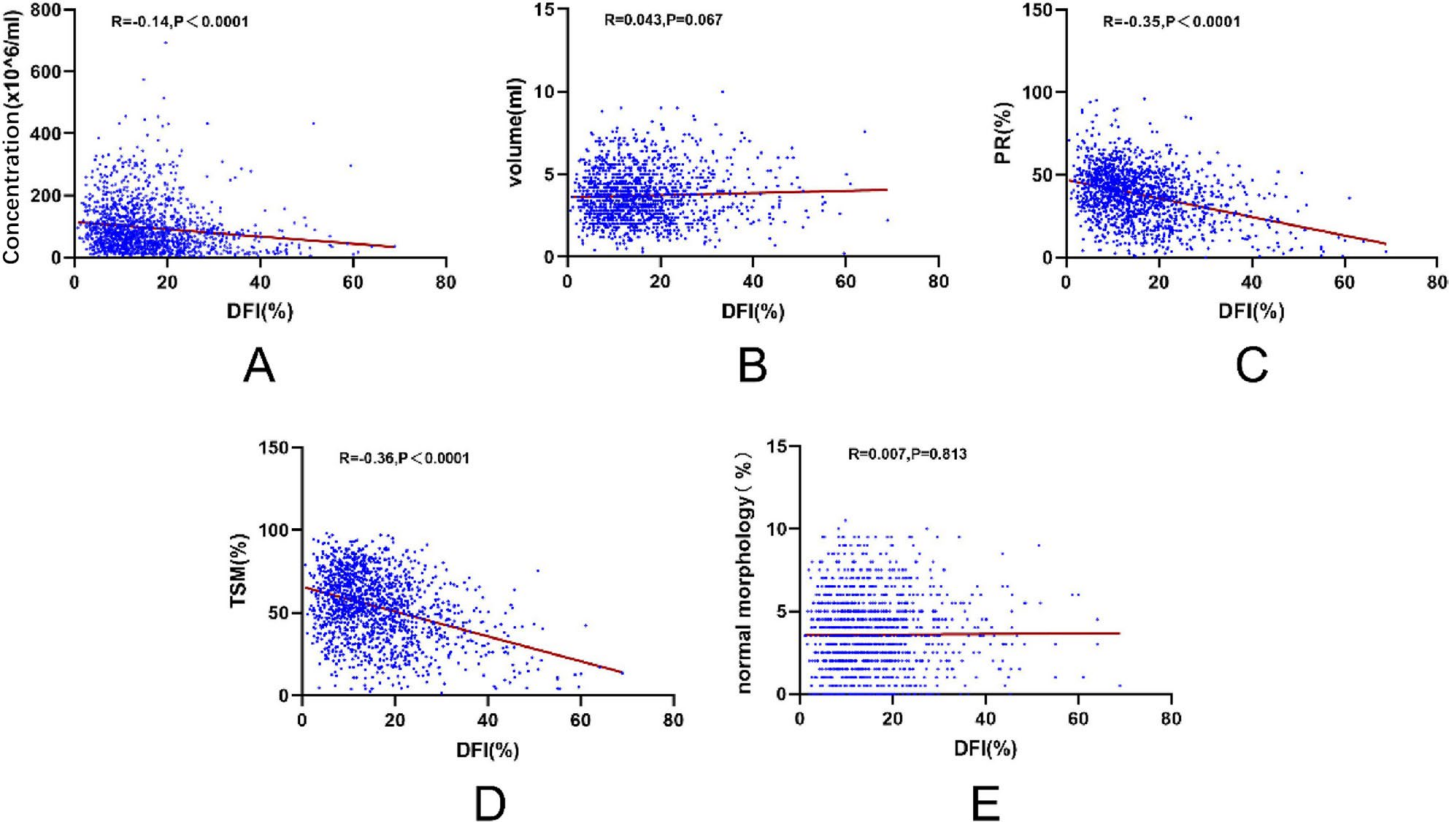
Simple linear regression between DFI and conventional sperm parameters **A** concentraction; **B** volume; **C** PR; **D **TSM; **E **normal morphology

To further explore the associations between sperm DFI and semen parameters (concentration, PR, TSM, and volume), simple linear regression analyses were conducted. The results revealed moderate negative correlations between DFI and PR (*R* = − 0.35, *P* < 0.0001) as well as TSM (*R* = − 0.36, *P* < 0.0001), a slight negative correlation with sperm concentration (*R* = − 0.14, *P* < 0.0001), and no significant correlation with either semen volume or sperm morphology (Fig. 2).

Multivariate linear regression was subsequently performed to adjust for potential confounders, including male age, varicocele status, and testicular volume. The results, presented in Table 2, confirmed that sperm DFI was independently associated with sperm concentration (*B*=-31.15, 95% CI: -44.75 to -17.55, *P* < 0.001), PR (*B*=-12.21, 95% CI: -14.87 to -9.56, *P* < 0.001), and TSM (*B*=-18.00, 95% CI: -21.38 to -14.62, *P* < 0.001). Regarding sperm morphology, the multivariate regression analysis based on the imputed dataset showed no significant association with DFI (B = 0.35, 95% CI: -0.29 to 1.00, *P* = 0.262).

**Table 2:** Multivariate Linear Regression Analysis of Associations Between Sperm DFI and Conventional Semen Parameters

Outcome	Group	B (95%CI)	t	*P*
Sperm concentration	DFI<30%^#^			
	DFI ≥ 30%	-31.15(-44.75~-17.55)	-4.49	< 0.001^*^
PR	DFI<30%^#^			
	DFI ≥ 30%	-12.21 (-14.87 ~ -9.56)	-9.03	< 0.001^*^
TSM	DFI<30%^#^			
	DFI ≥ 30%	-18.00 (-21.38 ~ -14.62)	-10.44	< 0.001^*^
Normal morphology	DFI<30%^#^			
	DFI ≥ 30%	0.35 (-0.29 ~ 1.00)	1.17	0.262

Adjusted for male age, varicocele status, and testicular volume

*DFI* DNA fragmentation index, *PR* progressive motility, *TSM* total sperm motility

^*^Indicates *P* < 0.05

^#^Reference

### Laboratory outcomes in IVF/ICSI cycles

Female baseline characteristics and ovarian stimulation parameters are compared in Table 3. No significant differences were observed in most indicators such as female BMI, baseline FSH, or LH levels. However, the high DFI group had significantly older female partners (35.96 vs. 33.98 years, *P* < 0.001), lower E_2_ levels on the trigger day (1689.53 vs. 2094.80 pg/mL, *P* = 0.026), and a lower proportion of IVF cycles (56.06% vs.79.54%, *P* < 0.001).

**Table 3:** Female baseline characteristics and ART cycle parameters stratified by DFI group

Variable	Total (*n* = 1784)	Normal DFI(<30%) (*n* = 1652)	High DFI (≥ 30%) (*n* = 132)	Statistic	*P*
Female age (year)	34.12 ± 5.25	33.98 ± 5.23	35.96 ± 5.19	t=-4.198	< 0.001^*^
Female BMI (kg/m^2^)	23.78 ± 3.75	23.79 ± 3.72	23.61 ± 4.05	t = 0.534	0.593
bFSH (mIU/mL)	7.10 (5.74–8.79)	7.09 (5.70–8.75)	7.24 (5.98–9.24)	Z = 1.300	0.194
bLH (mIU/mL)	4.48 (3.23–6.20)	4.47 (3.22–6.19)	4.72 (3.33–6.28)	Z = 0.786	0.432
bE_2_ (pg/mL)	38.29 (28.82–54.08)	38.29 (28.76–54.00)	38.38 (29.04–55.95)	Z = 0.724	0.469
bP_4_ (ng/mL)	0.49 (0.28–0.78)	0.48 (0.28–0.78)	0.50 (0.28–0.79)	Z = 0.373	0.709
Parity, n (%)				χ²=1.078	0.299
Primipara	1260 (70.63)	1172 (70.94)	88 (66.67)		
Multipara	524 (29.37)	480 (29.06)	44 (33.33)		
Ovulation induction protocol, n (%)				χ²=1.492	0.474
GnRH antagonist	1297 (72.7)	1207 (73.06)	90 (68.18)		
GnRH agonist	364 (20.4)	333 (20.16)	31 (23.48)		
others	123 (6.89)	112 (6.78)	11 (8.33)		
Gn duration(day)	9.51 ± 2.15	9.51 ± 2.11	9.57 ± 2.59	t=-0.323	0.747
Total Gn dose (IU)	2380.22 ± 885.80	2379.73 ± 883.08	2386.34 ± 922.62	t=-0.083	0.934
Number of Opu cycle	1.00 (1.00–1.00)	1.00 (1.00–1.00)	1.00 (1.00–1.00)	Z = 1.233	0.371
Trigger drug, n (%)				χ²=0.939	0.625
HCG	1511 (85.17)	1396 (84.97)	115 (87.79)		
HCG+ GnRH-a	238 (13.42)	223 (13.57)	15 (11.45)		
GnRH-a	25 (1.41)	24 (1.46)	1 (0.76)		
LH on trigger day (mIU/mL)	2.07 (1.17–3.73)	2.07 (1.17–3.76)	2.17 (1.21–3.59)	Z = 0.312	0.755
E_2_ on trigger day (pg/mL)	2050.00 (1160.67–3877.90)	2094.80 (1169.93–3934.00)	1689.53 (1121.35–2911.12)	Z = 2.219	0.026*
P_4_ on trigger day bP_4_ (ng/mL)	1.05 (0.71–1.47)	1.05 (0.71–1.47)	1.01 (0.67–1.41)	Z = 1.331	0.183
Number of retrieved oocytes(n)	9.00 (5.00–15.00)	9.00 (5.00–15.00)	9.00 (4.00–13.00)	Z = 1.579	0.115
Insemination mode, n (%)				χ²=39.018	< 0.001*
IVF	1388 (77.8)	1314 (79.54)	74 (56.06)		
ICSI	396 (22.2)	338 (20.46)	58 (43.94)		
Cycle result, n (%)				χ²=1.894	0.388
Freeze-all embryos	889 (49.83)	824 (49.88)	65 (49.24)		
Fresh-embryo transfer	736 (41.26)	685 (41.46)	51 (38.64)		
No available embryos	159 (8.91)	143 (8.66)	16 (12.12)		

*BMI* body mass index, *bFSH* baseline follicle-stimulating hormone, *bLH* baseline luteinizing hormone, *bE*_*2*_ baseline estradiol, *bP*_*4*_ baseline progesterone, *Gn* Gonadotropin, *Opu* ovum pick up, *HCG* human chorionic gonadotophin, *GnRH-a* GnRH agonist, *IVF* in vitro fertilization, *ICSI* intracytoplasmic sperm injection

^*^Indicates *P* < 0.05

As shown in Table 4, unadjusted 2PN fertilization rates showed no significant difference between groups (median [IQR]: 0.636 [0.476–0.800] vs. 0.592 [0.400–0.750], *P* = 0.072). However, after adjusting for female age, male age, E2 level on trigger day, and insemination mode in a beta regression model, high DFI was significantly associated with a reduced 2PN fertilization rate (β = − 0.257; 95% CI: − 0.491 to − 0.024; *P* = 0.031). No significant differences were found in the proportion of patients with ≥ 1 top-quality D3 embryo (57.1% vs. 49.2%, *P* = 0.097) or > 50% transferable embryos (66.0% vs. 67.4%, *P* = 0.809). Logistic regression models adjusting for the same covariates confirmed the absence of significant associations for both outcomes (OR = 0.89; 95% CI: 0.61–1.30; *P* = 0.547 and OR = 1.22; 95% CI: 0.83–1.81; *P* = 0.320, respectively).

**Table 4:** Embryological outcomes according to DFI group: unadjusted and adjusted analyses

Outcome	Normal DFI (<30%)	High DFI (≥ 30%)	*P* (unadj)	Adjusted Effect (OR/β)	95% CI	*P* (adj)
2PN fertilization rate	0.636 (0.476–0.800)	0.592 (0.400–0.750)	0.072	β = − 0.257	(–0.491, − 0.024)	0.031*
≥ 1 Top-quality embryos (D3)	943/1652 (57.1%)	65/132 (49.2%)	0.097	OR = 0.89	(0.61, 1.30)	0.547
> 50% Transferable embryos(D3)	1090/1652 (66.0%)	89/132 (67.4%)	0.809	OR = 1.22	(0.83, 1.81)	0.320

*P* (adj), adjusted for female age, male age, E_2_ on trigger day, and insemination mode

*DFI* DNA fragmentation index, *2PN* two-pronuclei

^*^Indicates *P* < 0.05

### Pregnancy outcomes in fresh embryo transfer cycles

Analysis of 736 fresh transfer cycles revealed that the high DFI group had older female (36.61 vs. 34.24 years, *P* < 0.001) and male partners (37.76 vs. 34.70 years, *P* = 0.003), lower PR (28.03% vs. 39.87%) and TSM (37.47% vs. 55.79%), and a higher proportion of ICSI cycles (41.18% vs. 20.15%). (Table 5). Unadjusted and adjusted comparisons of pregnancy outcomes showed no significant differences between groups for biochemical pregnancy, clinical pregnancy, live birth, pregnancy loss, or ectopic pregnancy (all *P* > 0.05) (Table 6).

**Table 5:** Baseline characteristics of patients in fresh embryo transfer cycles stratified by DFI groups

	Total(*n* = 736)	Group		statistic	*P*
		Normal (*n* = 685)	High (*n* = 51)		
Female age (year)	34.40 ± 4.98	34.24 ± 4.95	36.61 ± 4.90	t=-3.301	0.001*
Female BMI (kg/m^2^)	23.70 ± 3.72	23.77 ± 3.77	22.81 ± 2.85	t = 1.776	0.076
Gn duration(day)	9.30 ± 1.97	9.33 ± 1.99	8.96 ± 1.78	t = 1.289	0.198
Total Gn dose (IU)	2374.36 ± 840.02	2381.86 ± 843.76	2273.53 ± 789.02	t = 0.888	0.375
Male BMI (kg/m^2^)	26.21 ± 3.88	26.18 ± 3.90	26.65 ± 3.65	t=-0.820	0.413
Male age (year)	34.91 ± 5.60	34.70 ± 5.45	37.76 ± 6.78	t=-3.153	0.003*
Sperm volume(mL)	3.67 ± 1.51	3.65 ± 1.49	4.06 ± 1.69	t=-1.881	0.060
PR (%)	39.05 ± 15.40	39.87 ± 15.06	28.03 ± 15.77	t = 5.397	< 0.001*
TSM (%)	54.52 ± 19.84	55.79 ± 19.38	37.47 ± 18.18	t = 6.542	< 0.001*
Number of Opu cycles	1.00 (1.00–1.00)	1.00 (1.00–1.00)	1.00 (1.00–1.00)	Z=-0.156	0.876
Infertility duration (year)	3.00 (2.00–5.00)	3.00 (2.00–5.00)	3.00 (2.00–5.00)	Z=-1.339	0.180
Abstinence days (day)	4.00 (3.00–7.00)	4.00 (3.00–7.00)	5.00 (3.00–6.50)	Z=-0.614	0.539
Sperm concentration (x10^6^/mL)	73.55 (37.18–127.65)	77.09 (38.20–134.90)	48.10 (22.60–84.95)	Z=-3.530	< 0.001
LH on trigger day (mIU/mL)	2.26 (1.27–3.67)	2.24 (1.26–3.66)	2.39 (1.41–3.71)	Z=-0.258	0.796
E_2_ on trigger day (pg/mL)	1668.54 (1094.87–2457.11)	1688.83 (1099.99–2479.70)	1361.56 (979.71–2063.15)	Z=-1.888	0.059
P_4_ on trigger day(ng/mL)	0.92 (0.63–1.19)	0.92 (0.63–1.19)	0.90 (0.64–1.13)	Z=-0.448	0.654
Number of retrieved oocytes (n)	8.00 (5.00–11.00)	8.00 (5.00–11.00)	7.00 (4.00–10.00)	Z=-1.539	0.124
bFSH (mIU/mL)	7.25 (5.97–8.95)	7.28 (5.96–8.91)	6.97 (6.01–9.93)	Z=-0.371	0.711
bLH (mIU/mL)	4.09 (3.13–5.59)	4.09 (3.13–5.58)	4.06 (3.16–5.59)	Z=-0.225	0.822
bE_2_ (pg/mL)	37.26 (28.96–52.19)	37.13 (28.91–51.14)	37.73 (29.07–56.87)	Z=-0.619	0.536
bP_4_(ng/mL)	0.48 (0.29–0.73)	0.48 (0.29–0.73)	0.50 (0.34–0.75)	Z=-0.818	0.413
Ovulation induction protocol, n (%)				χ²=3.291	0.193
GnRH antagonist	599 (81.39)	553 (80.73)	46 (90.20)		
GnRH agonist	117 (15.9)	112 (16.35)	5 (9.80)		
others	20 (2.72)	20 (2.92)	0 (0.00)		
Trigger drug, n (%)				χ²=0.491	0.483
HCG	678 (92.62)	629 (92.36)	49 (96.08)		
HCG+ GnRH-a	54 (7.38)	52 (7.64)	2 (3.92)		
Infertility type, n (%)				χ²=0.552	0.457
Primary infertility	296 (40.22)	278 (40.58)	18 (35.29)		
Secondary infertility	440 (59.78)	407 (59.42)	33 (64.71)		
Insemination mode, n (%)				χ²=12.396	< 0.001*
IVF	577 (78.4)	547 (79.85)	30 (58.82)		
ICSI	159 (21.6)	138 (20.15)	21 (41.18)		
Type of embryos transferred, n (%)				^_##^	0.395
D3	684(92.9)	638(93.14)	46(90.20)		
D5	52(7.07)	47(6.86)	5(9.80)		
Number of embryos transferred, n (%)				χ²=0.735	0.391
1	207 (28.12)	190 (27.74)	17 (33.33)		
2	529 (71.88)	495 (72.26)	34 (66.67)		

*DFI* DNA fragmentation index, *BMI* body mass index, *bFSH* baseline follicle-stimulating hormone, *bLH* baseline luteinizing hormone, *bE*_*2*_ baseline estradiol, *bP*_*4*_ baseline progesterone, *Gn* Gonadotropin, *Opu* ovum pick up, *HCG* human chorionic gonadotophin, *GnRH-a* GnRH agonist, *IVF* in vitro fertilization, *ICSI* intracytoplasmic sperm injection, *2PN* two-pronuclei, *PR* progressive motility, *TSM* total sperm motility

^*^Indicates *P* < 0.05

^##^Fisher exact test

**Table 6:** Pregnancy outcomes in fresh embryo transfer cycles by DFI group: unadjusted and adjusted analyses

Outcome	Normal DFI (<30%)	High DFI (≥ 30%)	*P* (unadj)	Adjusted OR	95% CI	*P* (adj)
Biochemical pregnancy	305 (44.53)	21 (41.18)	0.642	1.06	(0.57, 1.95)	0.840
Clinical pregnancy	291 (42.48)	20 (39.22)	0.649	1.07	(0.57, 1.92)	0.820
Live birth	254 (37.08)	14 (27.45)	0.168	0.83	(0.42, 1.59)	0.590
Pregnancy loss†	37 (12.71)	6 (30.00)	0.067	2.17	(0.68, 6.22)	0.160
Ectopic pregnancy	14 (2.04)	1 (1.96)	> 0.999	1.07	(0.05, 6.68)	0.950

*P* (adj), adjusted for male age, female age, PR, sperm concentration, insemination mode

*DFI* DNA fragmentation index

### RCS analysis of DFI and ART outcomes

The RCS analysis revealed complex associations between continuous DFI and ART outcomes (Supplementary Figure S1). A marginally significant linear downward trend was observed for the 2PN fertilization rate (*P* total = 0.067, *P* non-linear = 0.994). Notably, a statistically significant non-linear relationship was identified for the live birth rate (*P* non-linear = 0.045), with the curve showing a steeper decline as DFI exceeded the 30% range. For pregnancy loss, although the overall association was not statistically significant, a visual upward trend in risk was observed in the high DFI range.

### Sensitivity analyses

**Fig. 3:**
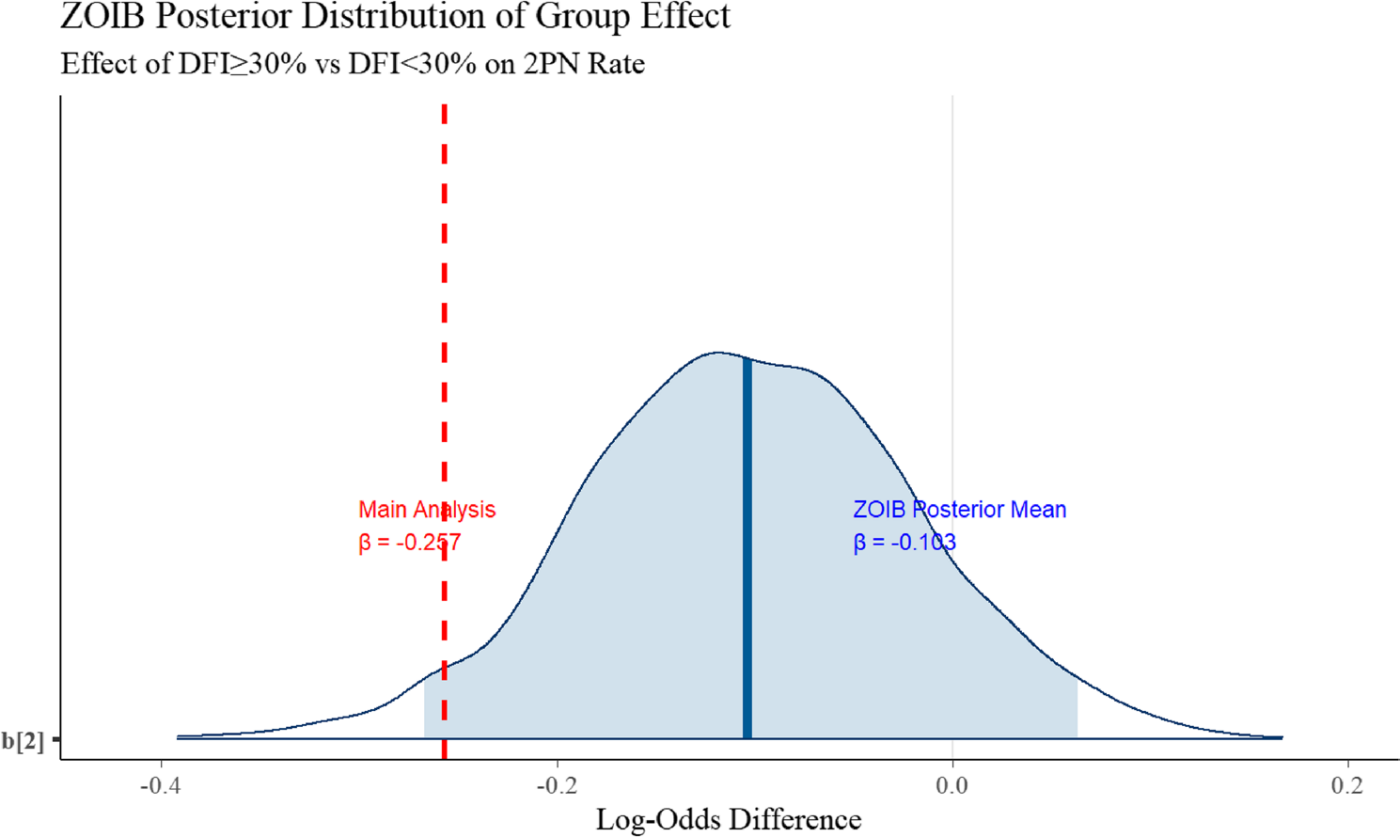
Sensitivity analysis of DFI ≥ 30% effect on 2PN rate using ZOIB regression

Sensitivity analyses largely supported the findings from the main analyses. As shown in Fig. 3, the posterior distribution (blue) of the effect of DFI ≥ 30% on the 2PN rate in the ZOIB model was concentrated in the negative region (posterior mean β = -0.103), indicating an 89.1% probability that high DFI reduces 2PN rate. However, this effect did not reach statistical significance (95% CI: -0.267 to 0.063). The main analysis result (red vertical line, β = -0.257) lies in the left tail of the ZOIB posterior distribution, suggesting that the data shrinkage approach may have overestimated the effect size.

**Fig. 4:**
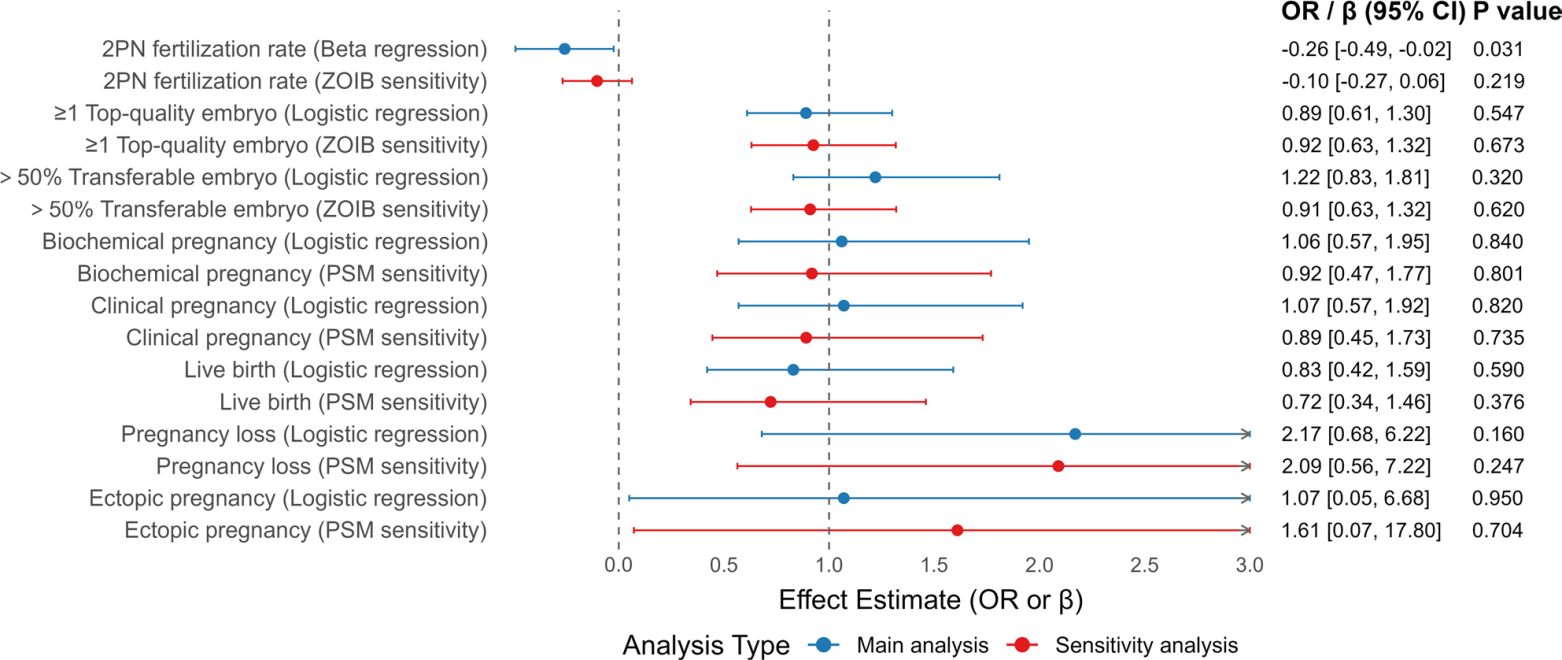
Forest plot comparing primary and sensitivity analyses for embryological and pregnancy outcomes

No significant associations were observed for other embryological outcomes (≥ 1 top-quality embryos, > 50% transferable embryo) or pregnancy outcomes, including biochemical pregnancy, clinical pregnancy, live birth, pregnancy loss, and ectopic pregnancy. A summary of effect estimates (β or OR) and 95% CIs from both main and sensitivity analyses is presented in Fig. 4.

Furthermore, sensitivity analyses using alternative DFI cut-offs (25% and 35%) yielded results completely consistent with the primary findings (Supplementary Table S2). High DFI remained significantly associated with a decreased 2PN fertilization rate (25% cut-off: β = -0.219, *P* = 0.013; 35% cut-off: β = -0.340, *P* = 0.018), while no significant impacts on clinical pregnancy or live birth were observed.

## Discussion

The analysis of 1784 IVF/ICSI cycles indicates a significant negative correlation between sperm DFI and conventional semen parameters including sperm concentration, PR, and TSM. These findings are in agreement with several previous studies [17–20]. Although Zhang et al. observed no correlation between DFI and sperm concentration or TSM, they did report a similar negative correlation between DFI and PR, supporting the robustness of our results [21]. Therefore, it appears that sperm DNA damage may be a crucial molecular contributor to asthenospermia. If DFI can be effectively reduced in such individuals, alleviating sperm DNA damage might help restore their reproductive potential.

However, the retrospective nature of the data limits the ability to infer causality. It remains possible that poor sperm motility adversely affects DFI rather than being a consequence of it. Alternatively, both motility defects and elevated DFI may reflect a shared underlying pathology, such as increased reactive oxygen species (ROS) levels [22] or mitochondrial damage [23], rather than one directly causing the other. Longitudinal or mechanistic studies are needed to elucidate the directionality of this relationship.

Although sperm morphology data exhibited a missing rate of approximately 29%, we confirmed that the missingness was random. Subsequent analysis using multiple imputation revealed no significant association between DFI and sperm morphology. Previous studies have shown inconsistent results regarding the relationship between DFI and morphology. Some have reported a significant negative correlation between DFI and the proportion of morphologically normal sperm [18, 24–26], while others found no significant association [17, 27]. One research further indicated that defects in the sperm head, tail, midpiece, and neck were all not significantly correlated with DFI [28]. The heterogeneity in findings may be attributed to differences in morphology assessment methods or laboratory protocols.

Our research indicates that elevated sperm DFI significantly associated with a reduced 2PN fertilization rate, while showing no significant impact on subsequent embryo development. These findings align with previous reports indicating that sperm DNA damage mainly affects fertilization. A recent study [29] observed similar results—DFI was associated with reduced fertilization, but not with other embryological parameters. In contrast, another study [30] found no associations between DFI and any embryological outcomes. As previously discussed, high DFI is closely linked to reduced sperm motility, and motility is a well-established determinant of fertilization potential [31, 32]. Therefore, it is plausible that increased DFI reflects broader defects in sperm function, thereby reducing the likelihood of successful fertilization. After fertilization, paternal DNA damage may be partially repaired by the oocyte or the fertilized egg, as all DNA repair in pre-implantation embryos relies on maternal contribution [33, 34]. This could explain why high DFI does not appear to significantly impair early embryo development in our study.

This study found that sperm DFI did not have a significant impact on clinical pregnancy rates, miscarriage rates, or live birth rates in fresh embryo transfer cycles. This may be attributed to the procedures such as density gradient centrifugation and upstream method applied to sperm prior to IVF/ICSI, which effectively removed a substantial number of sperm with poor motility. This selective process has likely diminished the impact of high sperm DFI on pregnancy outcomes. This finding aligns with reports from Liu [17], Green [19], and Chen [35]. Moreover, research by Chen et al. revealed that sperm DFI did not significantly affect neonatal outcomes, including sex, gestational age, preterm birth, or birth weight. Additionally, other studies have indicated that sperm DFI had no influence on the pregnancy outcomes of intrauterine insemination (IUI) cycles [36].

However, simultaneously, studies have reported a significant negative correlation between sperm DFI and miscarriage rates as well as newborn birth weights in IVF/ICSI-ET cycles [37]. Research conducted by Zhang et al. found that the sperm DFI in patients with a history of unexplained miscarriages was significantly lower than that of infertile males [21]. Zini et al. performed a systematic review and meta-analysis, which indicated that elevated sperm DFI is significantly associated with an increased risk of pregnancy loss following IVF and ICSI procedures [38]. Furthermore, one study suggested that abnormally elevated sperm DFI increases the early miscarriage rate in IVF-ET cycles, although there was no significant correlation found with early miscarriage rates in ICSI-ET cycles [39]. However, this study had a small sample size, encompassing only 61 cycle data.

The discrepancies in research findings may be attributed to variations in inclusion and exclusion criteria across studies, disparities in baseline data, the impact of confounding factors on clinical outcomes, the methodologies employed for sperm DFI testing, and the criteria used for categorizing sperm DFI. Consequently, this study attempted to minimize baseline heterogeneity and conducted regression adjustments for any divergent indicators. Additionally, the analysis of pregnancy outcomes was restricted to fresh embryo transfer cycles to minimize the confounding effects of frozen embryos and varying transfer protocols.

To overcome the limitations of arbitrary categorization, we further employed RCS analysis to explore the continuous relationship between DFI and ART outcomes. Interestingly, although our categorical analysis did not detect significant differences in live birth rates between the two groups, the RCS analysis provided a more granular insight, revealing a significant non-linear inflection point (*P* non-linear = 0.045). The curve indicates that the probability of live birth remains relatively stable in the lower DFI range but drops sharply once DFI exceeds the 30% threshold. This “threshold effect” may explain why simple binary comparisons often fail to capture significance if the high-risk group is diluted by borderline cases. Furthermore, although not statistically significant, the miscarriage risk curve exhibited a distinct upward trend beyond the 30% mark, visually corroborating the detrimental impact of high DFI on pregnancy maintenance. These findings support the clinical utility of the 30% cut-off recommended by previous SCSA studies [40–42], as it effectively captures the specific population at escalated risk.

From a methodological standpoint, we found that the unadjusted comparison for the 2PN fertilization rate showed only borderline significance. However, after adjusting for key confounders, beta regression revealed a statistically significant association with DFI. This may be partly due to the ability of multivariate modeling to reduce residual variance, but also to the fact that non-parametric tests, used in unadjusted analysis, generally have lower statistical power and do not fully exploit the continuous nature of the data. This demonstrates how residual variability may mask true effects in unadjusted analyses and highlights the importance of using appropriate statistical approaches as well as controlling for known covariates in reproductive outcome studies.

Although differing in statistical significance (main analysis *p* = 0.031 vs. sensitivity analysis ZOIB P(β < 0) = 89.1%), both analyses consistently demonstrate that DFI ≥ 30% tends to reduce 2PN rate. This discrepancy likely stems from the ZOIB model’s more rigorous handling of proportional data boundaries (0 and 1). Synthesizing both results, we conclude DFI ≥ 30% may modestly reduce 2PN rate, though further large-scale prospective studies are warranted to refine effect estimates.

Given that the 2PN fertilization rate is a bounded proportion variable (0–1), beta regression was preferred over linear regression to better accommodate its non-normal distribution. A standard shrinkage transformation was applied to account for boundary values. For D3 top-quality embryo and transferable embryo rates, due to high clustering at 0 or 1, variables were dichotomized and analyzed via logistic regression for interpretability.

In several previous studies, laboratory outcomes such as the 2PN fertilization rate and embryo development rates were calculated by dividing the total number of events by the total number of oocytes or embryos in each group. For instance, both Fu et al. [30] and Zhang et al. [39] used this group-level approach and conducted chi-square tests to compare proportions. While this method provides a general overview, it treats individual oocytes or embryos as independent observations, violating the independence assumption of chi-square testing. More importantly, it prevents the incorporation of patient-level variables (e.g. age) into statistical models, thereby limiting the ability to control for potential confounders—a common issue in many reports on this topic.

In contrast, Jiang et al. [29] calculated outcome rates at the individual patient level, treating each cycle as the unit of analysis. However, their study relied solely on Spearman correlation without conducting multivariable regression analysis. In our study, we not only adopted a patient-level analytical framework but also employed regression models appropriate for the distribution and nature of each outcome, while adjusting for key confounders from both male and female characteristics as well as features of the ART cycle, such as trigger-day hormone levels and insemination method. In addition, the robustness of our findings was further supported by sensitivity analyses tailored to outcome types. Moreover, the use of real-world clinical data enhances the external validity of our results.

Nonetheless, this study also has several limitations. First, due to the relatively small number of ICSI cycles, subgroup analyses by insemination method were not performed, although insemination mode was included as a covariate in multivariable models. Second, although emerging evidence suggests that recurrent miscarriages or repeated implantation failures may be associated with elevated DFI [43], we excluded those patients from this study. This decision was made to prioritize internal validity by minimizing confounding from potential female factors (e.g., immunologic or endometrial issues), which could have independently influenced pregnancy outcomes. However, we acknowledge that this rigorous selection limits the external validity of our findings, as it may exclude a high-risk subpopulation where DFI exerts a stronger effect. Third, the sample size of the high DFI group in the fresh embryo transfer cohort was relatively small. This limitation may have constrained statistical power, particularly for low-frequency events such as miscarriage. The limited number of events relative to the number of covariates also introduces a potential risk of overfitting in the regression model. Therefore, findings regarding clinical outcomes should be interpreted with caution. Finally, we did not assess blastocyst formation rates as an outcome, as nearly half of the cycles did not undergo extended embryo culture. Since the decision to proceed to blastocyst stage was largely based on embryo quality and number, including only selected cycles may have introduced selection bias.

## Conclusions

In summary, sperm DFI is associated with conventional semen parameters and appears to negatively affect fertilization, although no significant association with downstream embryonic or clinical outcomes was observed in our cohort. Future investigations integrating molecular mechanisms with rigorous statistical modeling may better define the role of sperm DFI in assisted reproduction.

## Supplementary Information

Supplementary Material 1: Figure S1. Restricted cubic spline (RCS) analysis of the association between sperm DNA fragmentation index (DFI) and reproductive outcomes.

## Data Availability

The datasets analyzed during the current study are available from the corresponding author upon reasonable request.
