# Supplementary material for: Association of sperm DNA fragmentation index with semen quality and ART outcomes: real-world evidence from a retrospective cohort
Source: Basic Clin Androl. 2026 Mar 20;36:9. doi: 10.1186/s12610-026-00308-2 (PMC13003730; doi:10.1186/s12610-026-00308-2)

Supplementary table S1

**Comparison of demographic and semen parameters between patients with and without available sperm morphology data**

| Variables | Morphology missing (n = 518) | Morphology available (n = 1266) | Statistic | *P* |
| --- | --- | --- | --- | --- |
|  |  |  |  |  |
| DFI (%) | 14.03 (9.28, 20.05) | 13.50 (9.00, 19.98) | Z=-0.50 | 0.614 |
| Male age (year) | 34.00 (31.00, 38.00) | 34.00 (31.00, 38.00) | Z=-0.42 | 0.678 |
| Sperm volume (mL) | 3.60 (2.80, 4.60) | 3.60 (2.60, 4.55) | Z=-1.40 | 0.163 |
| Sperm concentration (x10^6^/mL) | 73.19 (42.10, 118.85) | 75.69 (39.40, 128.19) | Z=-0.43 | 0.671 |
| NP (%) | 13.00 (7.73, 21.00) | 13.30 (7.70, 22.40) | Z=-0.40 | 0.689 |
| PR (%) | 38.29 (29.00, 47.77) | 38.79 (27.70, 48.80) | Z=-0.05 | 0.959 |

Z: Mann-Whitney test

Supplementary table S2

**Sensitivity analyses of main ART outcomes using alternative DFI cut-offs (25% and 35%)**

| Outcomes | DFI cut-off: 25% | | | DFI cut-off: 35% | | |
| --- | --- | --- | --- | --- | --- | --- |
|  | β/OR | 95%CI | *P* | β/OR | 95%CI | *P* |
| 2PN fertilization rate^a^ | -0.219 | (-0.391, -0.047) | 0.013^*^ | -0.340 | (-0.622, -0.058) | 0.018^*^ |
| Clinical pregnancy^b^ | 1.226 | (0.765,1.964) | 0.397 | 1.361 | (0.585, 3.163) | 0.474 |
| Live birth^b^ | 1.058 | (0.645, 1.735) | 0.824 | 0.731 | (0.278, 1.923) | 0.525 |

^*^Indicates P < 0.05

ᵃ Analyzed using Beta regression, adjusted for female age, male age, E2 on trigger day, and insemination mode.

ᵇ Analyzed using multivariable logistic regression among fresh embryo transfer cycles, adjusted for male age, female age, PR, sperm concentration, and insemination mode.

Supplementary figure S1.

Restricted cubic spline (RCS) analysis of the association between sperm DNA fragmentation index (DFI) and reproductive outcomes.


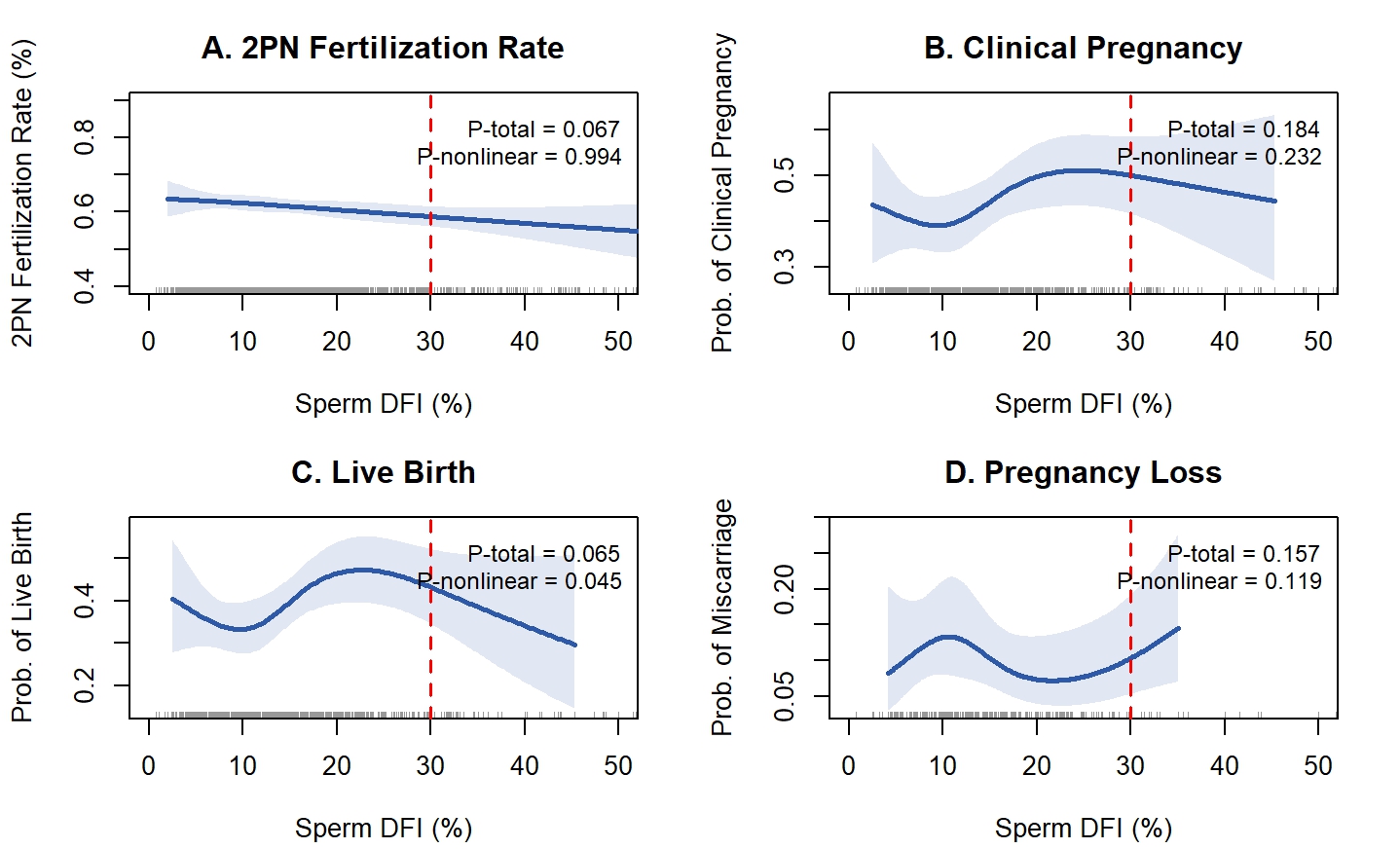

Supplement: Supplementary file 1 — Supplementary Material 1: Figure S1. Restricted cubic spline (RCS) analysis of the association between sperm DNA fragmentation index (DFI) and reproductive outcomes. [file 12610_2026_308_MOESM1_ESM.docx]
